# Correction: Plasma microRNAs biomarkers in mild cognitive impairment among patients with type 2 diabetes mellitus

**DOI:** 10.1371/journal.pone.0243177

**Published:** 2020-11-25

**Authors:** Iman I. Salama, Samia M. Sami, Ghada A. Abdellatif, Amira Mohsen, Hanaa Rasmy, Solaf Ahmed Kamel, Mona Hamed Ibrahim, Mona M.F. Ganem, Walaa A. Fouad, Hala M. Raslan

The eighth author's name is spelled incorrectly. The correct name is: Mona M.F. Ganem.
